# A Deep Learning Algorithm to Predict Hazardous Drinkers and the Severity of Alcohol-Related Problems Using K-NHANES

**DOI:** 10.3389/fpsyt.2021.684406

**Published:** 2021-07-09

**Authors:** Suk-Young Kim, Taesung Park, Kwonyoung Kim, Jihoon Oh, Yoonjae Park, Dai-Jin Kim

**Affiliations:** ^1^Department of Psychiatry, College of Medicine, The Catholic University of Korea, Seoul, South Korea; ^2^Department of Electrical Engineering and Computer Sciences, University of California, Berkeley, CA, United States; ^3^School of Electrical and Electronic Engineering, Yonsei University, Seoul, South Korea; ^4^Department of Electrical and Computer Engineering, Seoul National University, Seoul, South Korea

**Keywords:** machine learning, deep learning, hazardous drinkers, hazardous drinking, alcohol related problems, alcohol use disorder, alcohol dependence, K-NHANES

## Abstract

**Purpose:** The number of patients with alcohol-related problems is steadily increasing. A large-scale survey of alcohol-related problems has been conducted. However, studies that predict hazardous drinkers and identify which factors contribute to the prediction are limited. Thus, the purpose of this study was to predict hazardous drinkers and the severity of alcohol-related problems of patients using a deep learning algorithm based on a large-scale survey data.

**Materials and Methods:** Datasets of National Health and Nutrition Examination Survey of South Korea (K-NHANES), a nationally representative survey for the entire South Korean population, were used to train deep learning and conventional machine learning algorithms. Datasets from 69,187 and 45,672 participants were used to predict hazardous drinkers and the severity of alcohol-related problems, respectively. Based on the degree of contribution of each variable to deep learning, it was possible to determine which variable contributed significantly to the prediction of hazardous drinkers.

**Results:** Deep learning showed the higher performance than conventional machine learning algorithms. It predicted hazardous drinkers with an AUC (Area under the receiver operating characteristic curve) of 0.870 (Logistic regression: 0.858, Linear SVM: 0.849, Random forest classifier: 0.810, K-nearest neighbors: 0.740). Among 325 variables for predicting hazardous drinkers, energy intake was a factor showing the greatest contribution to the prediction, followed by carbohydrate intake. Participants were classified into Zone I, Zone II, Zone III, and Zone IV based on the degree of alcohol-related problems, showing AUCs of 0.881, 0.774, 0.853, and 0.879, respectively.

**Conclusion:** Hazardous drinking groups could be effectively predicted and individuals could be classified according to the degree of alcohol-related problems using a deep learning algorithm. This algorithm could be used to screen people who need treatment for alcohol-related problems among the general population or hospital visitors.

## Introduction

Health problems associated with alcohol-related diseases are becoming prevalent worldwide (1). Common and mild alcohol related diseases tend to ease in adolescence. However, more serious diseases can become chronic and require long-term medical and psychological management (2). Alcohol-related disorders can cause significant disabilities. They are associated with many physical and mental illnesses (3–5). Alcohol-related disorders share similar clinical course and are associated with substantially increased morbidity and mortality (6, 7). Because of these risks, large-scale studies have been conducted in many countries for decades to identify and predict risk factors, prevalence, and prognosis of alcohol use disorders.

Although previous studies have warned dangers of alcohol, patients who are dependent on alcohol or addicted to alcohol rarely visit the hospital due to the lack of insight and the underestimation of urgency of treatment (8). In addition, patients might be hesitant to seek treatment due to stigma surrounding mental illness including alcohol-related disorders. One study found that patients with a higher stigma for alcohol-related disorders were more unlikely seek treatment (9). Most patients with alcohol problems start to search for clinical help only when they have serious complications such as alcoholic hepatitis, cardiovascular disease, or gastrointestinal cancer. In the United States, only 13% of alcohol-dependent patients receive specialized treatment and only 24% of them seek some kind of help (10).

In addition, it is important to intervene at an early stage to minimize alcohol-related damage to patients with alcohol-related problems. To mediate prior to harmful consequences of alcohol drinking, the concept of hazardous drinking as an early stage of alcoholism has been introduced (11). Hazardous drinking is defined as the pattern of alcohol consumption that places patients at risk of adverse health events (12). The recent longitudinal study reported that after 6 years of follow-up of individuals who were hazardous drinkers, 46.9% of them still had alcohol use disorder and 15.4% of them became even more severe (13). Also, hazardous drinkers were more likely to have hypertension, diabetes, and COPD than the abstainers after the follow-up period (13). However, if hazardous drinkers were identified and intervened early, these risks could be reduced with relatively little effort. In one study, hazardous drinkers were offered regular telephone counseling for 1 year (14). As a result, about 5% of hazardous drinkers stopped drinking alcohol, and about 30% were reclassified as low-risk drinkers at the endpoint (14). To screen hazardous drinkers and identify them early, studies have been conducted on which demographic, social, and biological predictors of alcohol use disorder can contribute to alcohol-related problems.

Recently, deep learning has been actively used to screen and predict psychiatric diseases based on these predictors (15). A recent study has effectively identified patients with depression based on data of a large-scale survey from the United States, the National Health and Nutrition Examination Survey (16). Another study has also identified anxious and depressive participants using socio-demographic, occupational, and health-related information (17). On the other hand, early and mid-stage alcohol dependent patients with habitual drinking are often unaware of serious consequences of their alcohol dependence. If these unrecognized patients who normally receive primary care, health check-ups, and outpatient care other than psychiatry can be screened by a deep learning algorithm using predictors, intervention and management could take place at an earlier time to slow down the progression of diseases. However, to the best of our knowledge, no research has demonstrated the use of deep learning for screening and predicting hazardous drinkers based on large-scale data until now.

Large-scale surveys for disease prevalence and risk factor analysis have been conducted in various countries. The Korea National Health and Nutrition Examination Survey (K-NHANES) has been conducted every year in Korea. In this survey, participants were asked detailed questions regarding their alcohol-related problems, which allowed them to be classified into one of the four levels: Zone I, Zone II, Zone III, and Zone IV (12). If deep learning could predict the severity of these problems, it can be used as a reference for determining whether a patient needs hospitalization and the length of treatment.

In this study, we predicted hazardous drinkers in a large survey dataset through a deep learning algorithm and determined which factors contributed more to the deep learning process. In addition, performances of machine learning techniques other than deep learning such as support vector machine, logistic regression, and K-nearest neighbors were determined and compared to the performance of deep learning. We also determined whether deep learning could accurately predict the severity of alcohol-related problems in one of four severity levels.

## Materials and Methods

### Datasets

Datasets of National Health and Nutrition Examination Survey of South Korea (K-NHANES), a nationally representative survey with a complex, multi-stage stratification sample design for the entire South Korean population, were utilized to train deep learning. The K-NHANES is a national-wide survey conducted by the Division of Chronic Disease Surveillance, Korea Centers for Disease Control and Prevention since 1998 (18). Data of the K-NHANES IV (surveyed from 2007 to 2009), V (surveyed from 2010 to 2012), VI (surveyed from 2013 to 2015), and VII (surveyed from 2016 to 2018) were used. A professional survey team of Korea Centers for Disease Control and Prevention was formed to conduct annual surveys that could produce statistics every year without a seasonal bias. Among 303,180 geographically defined sampling units, 200 (K-NHANES IV) or 192 (K-NHANES V, VI, VII) primary units (PSUs) were sampled considering administrative districts and housing types. A total of 23 (K-NHANES IV, VII) or 20 (K-NHANES V, VI) households were systemically selected with intra-stratification of age, gender, and residential area from each PSU which contained 60 households on average. Nearly 10,000 individuals aged 1 year or more were targeted for K-NHANES. Subjectswere then divided into three groups according to their stage of life: children (aged 1~11 years), adolescents (aged 12~18 years), and adults (aged 19 years and over). Appropriate survey categories were then applied. K-NHANES not only collected questionnaire data such as demographic characteristics and dietary habits, but also obtained medical examination data using various laboratory tests.

**Figure 1:**
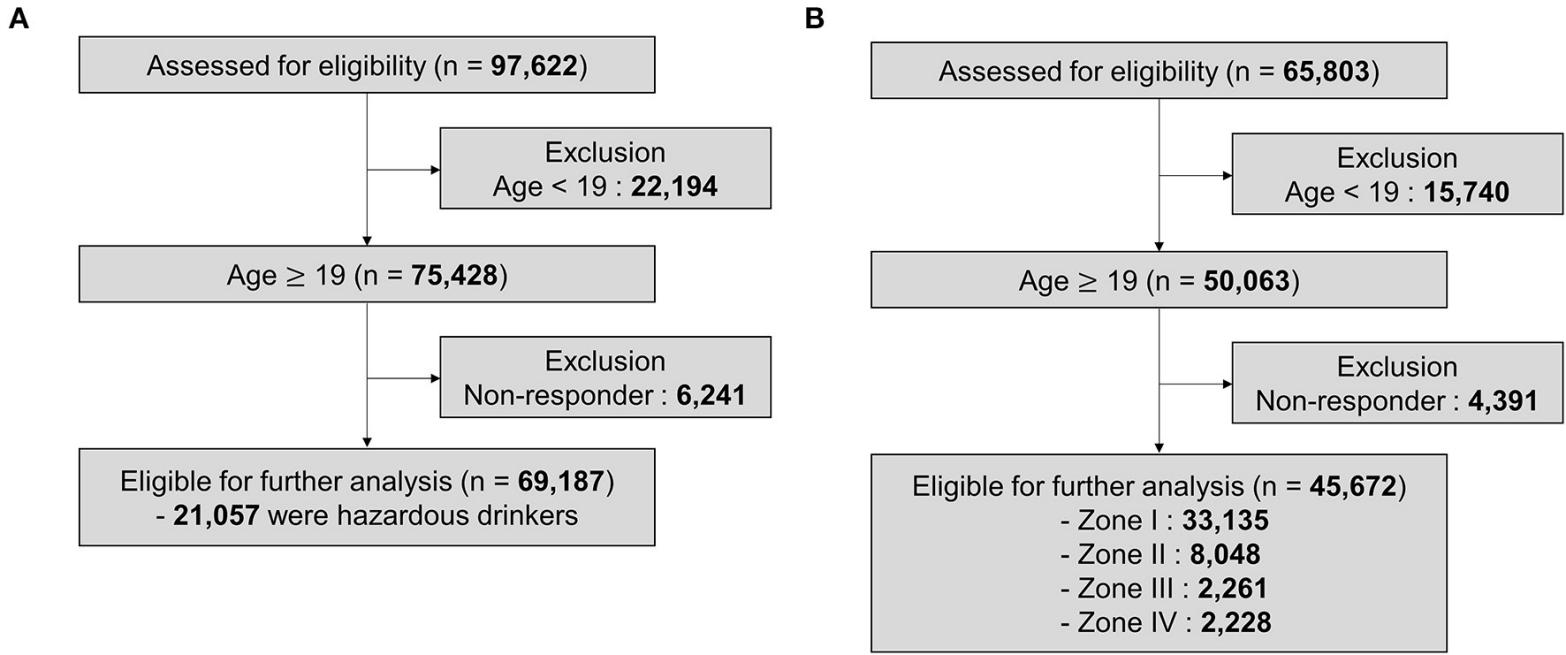
Profile of the study population. **(A)** K-NHANES dataset for predicting hazardous drinkers. **(B)** K-NHANES dataset for predicting the severity of alcohol-related problems.

In the analysis of K-NHANES, we included all variables from datasets. Initially, to make a model for predicting hazardous drinkers, we had 97,622 individuals and 795 variables from K-NHANES IV, V, VI, and VII. Of these 97,622 individuals, only those aged 19 years or more who responded appropriately to questions of Alcohol Use Disorders Identification Test (AUDIT) were analyzed. Variables directly related to alcohol use (e.g., monthly drinking rate, experience of driving under the influence of alcohol in the past 1 year) were excluded. Also, values of “9,” “99,” “999,” and “9999” meaning “I don't know about that question” for continuous variables were regarded as missing values because these values could mislead the prediction. In addition, variables created by statistical need, such as weights of variable and estimation of variance, were deleted. Variables with more than 18% of missing values were also excluded. As a result, 325 of 795 variables were utilized to build the deep learning model. These variables are listed in Supplementary Table 1. Therefore, 69,187 participants and 325 variables were used in the analysis to predict the hazardous drinking group (Figure 1A).

To establish a model for predicting the severity of alcohol-related problems, only data that asked all 10 questions of AUDIT among K-NHANES could be used. In some survey years, only four questions rather than all 10 questions were examined in a simplified form. Although these four questions could distinguish hazardous drinkers, they were difficult to classify severities. Therefore, we created a model to predict the severity using datasets of K-NHANES IV, V, and a part of VI (2013, 2015) that investigated all 10 questions. Initially, 65,803 participants were extracted from the above datasets. Of them, only those aged 19 years or more who responded correctly to AUDIT questions were used in the analysis. We removed alcohol related variables and regarded non-response values as missing values. We then excluded variables with more than 25% missing values. Thus, a total of 392 variables were used for the final analysis (Supplementary Table 2). Finally, 45,672 individuals and 392 variables were analyzed to make the model for predicting the severity of alcohol-related problems (Figure 1B).

### Evaluation of Alcohol Use

Alcohol Use Disorder Identification Test (AUDIT) is one of the most reliable screening tools to test whether someone has an alcohol-related problems (12). In 1989, World Health Organization devised the AUDIT to screen patients with hazardous drinking, harmful drinking, and alcohol dependence. It consists of 10 questions to inquire about quantity and frequency of drinking (Question 1–3, consumption score), symptom of dependence (Question 4–6, dependent score), and alcohol-related problems caused by harmful drinking (Question 7–10, alcohol-related problem score). Recent studies have emphasized that AUDIT is more effective than CAGE questionnaire (Cut down, Annoyed, Guilty, and Eye opener), a previously used test, for diagnosing hazardous drinking, alcohol abuse, and alcohol dependence (19).

To be used in various situations, there are many abbreviated versions of AUDIT, including AUDIT-QF (only contains Questions 1 and 2 of AUDIT), AUDIT-C (Questions 1, 2, 3), AUDIT-4 (Questions 1, 2, 3, 10) (20–27). Since the start of K-NHANES, not all AUDIT questions have always been included in K-NHANES. K-NHANES IV, V, and a part of VI (2013, 2015) included all AUDIT questions in the survey. However, K-NHANES VII and a part of K-NHANES VI (2014) used AUDIT-4 (Questions 1, 2, 3, 10), a simplified version of AUDIT. The abbreviated version of AUDIT had the advantage of being simple. It could classify hazardous drinkers. However, it could not classify the severity of alcohol-related problems. Only the full version of the AUDIT could divide the severity of alcohol problems into four stages: Zone I, Zone II, Zone III, and Zone IV. Zone IV is the most serious condition. Details such as the cut off value of each test are described in Supplementary Methods (28, 29).

In summary, K-NHANES IV (2007–2009), V (2010–2012), VI (2013–2015), and VII (2016–2018) were used to predict hazardous drinkers. However, only K-NHANES IV, V, and a part of VI (2013, 2015) that employed full AUDIT questions were used to predict the severity of alcohol-related problems.

### Model Development and Validation

Our deep neural network consisted of the following architectural designs. For each input variable as an encoded response to a survey question, a dense layer was applied and then L2-normalized to project the input onto an eight-dimensional unit hypersphere. All embeddings were then concatenated along the channel dimension to produce an aggregated representation, which was then passed to a multi-layer perceptron (MLP) of several layers to produce the final prediction.

We tried various combinations of hidden layers. We changed the number of hidden layers from 1 to 6. The number of nodes per layer was changed from 4,096 to 2,048, 1,024, and 512 to try various combinations. As a result, in the model predicting hazardous drinkers, the maximal area under the receiver operating characteristic curve (AUC) was derived when the hidden dimension of the MLP were set to be “2,048, 2,048, 2,048, and 512.” In addition, in the model predicting the severity of alcohol problem, optimal results were derived when the MLP was set to be “2,048, 2,048, 2,048, 2,048, 2,048, and 512.” At each layer, we also applied batch normalization (30), Swish activation (31), and dropout with 50% chance (32).

We used an SGD optimizer with an initial learning rate of 0.2, 0.1, or 0.05, a Nesterov momentum of 0.9, 0.99, or 0.999 (33), and a weight decaying at the rate of 10^−6^. At each epoch, the learning rate was decayed by a factor of 0.999. The model was trained up to 50 epochs and the batch size was 4,096. Hyperparameters were chosen based on a 10-fold cross validation on the entire dataset (34). We also reported our evaluation metrics on the 10-fold cross validation. At test time, the dropout was turned off. Training and evaluation of the deep learning model were performed using Pytorch (35).

Various algorithms of conventional machine learning were used to compare their performances of deep learning, including logistic regression, support vector machine, random forest classifier, and K-nearest neighbors. Logistic regression is a type of regression analysis technique that could be performed especially when the dependent variable was binary among regression analysis for predictive analysis (36). Support vector machine (SVM) is one of techniques for finding a group classification rule for a given sample group (37). Random forest classifier is a type of ensemble learning algorithm used for classification and regression analysis. It is operated by outputting a classification or average predicted value from decision trees constructed during a training process (38). K-nearest neighbors is a methodology that can predict new data using information from the k nearest neighbors among existing data when new data are given (39). In some algorithms, machine learning was performed while changing the parameters, of which parameters showing maximum performance were adopted. These parameters used for machine learning are described in Supplementary Table 3.

We verified all algorithms through 10-fold cross validation. In addition, performances of deep learning and conventional machine learnings were compared using 10 sub-datasets obtained through 10-fold cross validation. One-way analysis of variance (ANOVA) and *post-hoc* test were then performed to determine whether deep learning showed significantly better performance than each conventional machine learning algorithm (Supplementary Table 4).

### Contribution-Ranking Analysis of Variables

There are several ways to obtain the contribution of each variable. Among them, to measure the significance of each survey question used by the trained model, we studied the drop-in performance when the response to a question was removed. A significant drop indicated that the question was serving as a strong cue for making the prediction by the model. Note that our model was in fact trained to deal with missing inputs due to dropout within the model. We summarized and sorted the observed gap of all input variables in Supplementary Table 1.

## Result

### Characteristics of K-NHANES Dataset for Predicting Hazardous Drinkers

How the subjects used in deep learning analysis were classified was summarized in Figure 1. To classify hazardous drinkers, we extracted data from K-NHANES IV, V, VI, VII surveyed from 2007 to 2018. The total number of participants was initially 97,622. Among them, 22,194 were excluded because they were under 19 years old. Then 6,241 individuals were excluded because they did not respond to questions about alcohol behavior. Finally, 69,187 participants were used in the machine learning model, of which 21,057 (30.4%) participants were classified as hazardous drinkers (Figure 1A).

### Characteristics of K-NHANES Dataset for Predicting the Severity of Alcohol-Related Problems

To establish a model for predicting the severity of alcohol-related problems, we extracted data from K-NHANES IV, V, and a part of VI (2013, 2015) investigated from 2007 to 2015 except for 2014. Total number of participants from the dataset was 65,803. Of them, 15,740 participants were excluded because they were under 19 years old. Then 4,391 individuals were excluded because they did not answer questions related to alcohol. The remaining 45,672 participants were used for machine learning modeling, including 33,135 (72.5%) in Zone I, 8,048 (17.6%) in Zone II, 2,261 (5.0%) in Zone III, and 2,228 (4.9%) in Zone IV (Figure 1B).

### Predicting Hazardous Drinkers in K-NHANES Dataset

**Figure 2:**
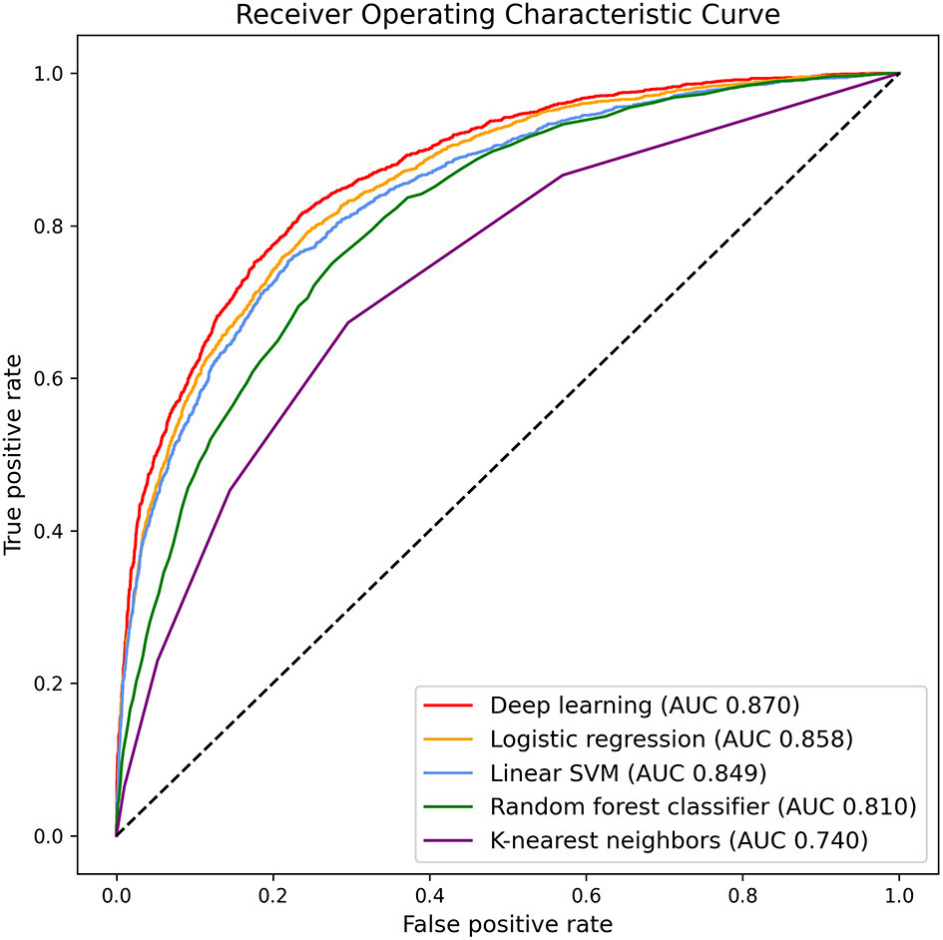
Area under the receiver operating characteristic curve for predicting hazardous drinkers in K-NHANES.

Deep learning and other conventional machine learning algorithms were trained with 325 variables and 69,187 subjects to predict hazardous drinkers in the dataset representing the general population. The performance of each model was evaluated with an area under the receiver operating characteristic curve (AUC) resulting from a 10-fold cross validation. Results are summarized in Figure 2. Deep learning showed the highest performance, predicting hazardous drinkers with an AUC of 0.870, followed by logistic regression, linear support vector machine, random forest classifier, and K-nearest neighbors, with AUC of 0.858, 0.849, 0.810, and 0.740, respectively. Detailed parameters of conventional machine learning algorithms are described in Supplementary Table 1.

There were significant differences in classification performance among algorithms based on AUC (one-way ANOVA, *F* = 1606.0, *p* < 0.001). Additionally, *post-hoc* analysis confirmed that deep learning was significantly superior to logistic regression (*p* < 0.001), linear support vector machine (*p* < 0.001), random forest classifier (*p* < 0.001), and K-nearest neighbors (*p* < 0.001, Supplementary Table 4).

We also calculated other measures of classifiers. Accuracy, precision, true positive rate, false positive rate, and F1-score of deep learning were 0.822, 0.756, 0.624, 0.090, and 0.684, respectively. Measures of other classifiers are described in Supplementary Table 5.

### Contribution of Each Variable to the Prediction of Hazardous Drinkers

We calculated the contribution of each variable based on the decrease of AUC when each variable was deleted for learning. It was considered that the greater the decrease in AUC when deep learning excluding a variable, the greater the contribution of that variable to the prediction from deep learning. Table 1 summarizes the ranking of contributions of top 20 variables. Supplementary Table 1 lists all variables by contribution. Among 325 variables, energy intake was found to be a factor with the greatest contribution to the prediction. Excluding this variable, the AUC of the trained model was only 0.618, which was the lowest AUC value. The second factor with the largest contribution was carbohydrate intake. After excluding this factor, the accuracy of the model was only 0.778 based on AUC. Among categorical variables, sex, lifelong smoking, and current smoking were ranked the 6th, the 13th, and the 16th, respectively, among all variables.

**Table 1:** Contribution ranking of top 20 variables used to predict hazardous drinkers in K-NHANES.

**Contribution ranking**	**Variable code**	**Variable description**	**AUC obtained by excluding this variable**
1	N_EN	Energy intake (Kcal)	0.6179801
2	N_CHO	Carbohydrate intake (g)	0.7757575
3	N_FAT	Fat intake (g)	0.8372522
4	age	Age	0.8387202
5	HE_HDL_st2	HDL-cholesterol	0.8564337
6	sex	Sex	0.856489
7	N_PROT	Protein intake (g)	0.8607539
8	HE_RBC	Red blood cells	0.8614541
9	HE_TG	Triglyceride	0.8624155
10	HE_ast	Aspartate aminotransferase	0.8633011
11	HE_alt	Alanine aminotransferase	0.8653823
12	HE_HB	Hemoglobin	0.8653889
13	BS1_1	(Adult) Lifetime smoking	0.8667786
14	HE_wc	Waist circumference	0.8674219
15	HE_chol	Total cholesterol	0.8681976
16	BS3_1	Current smoking status	0.8683784
17	N_INTK	Dietary intake (g)	0.8683819
18	HE_HCT	Hematocrit	0.8689445
19	HE_BMI	Body mass index	0.8692696
20	edu	Education level reclassification code	0.8693084

### Predicting Hazardous Drinkers by Top Ranked Variables or by Variables Related to Medical Records

**Figure 3:**
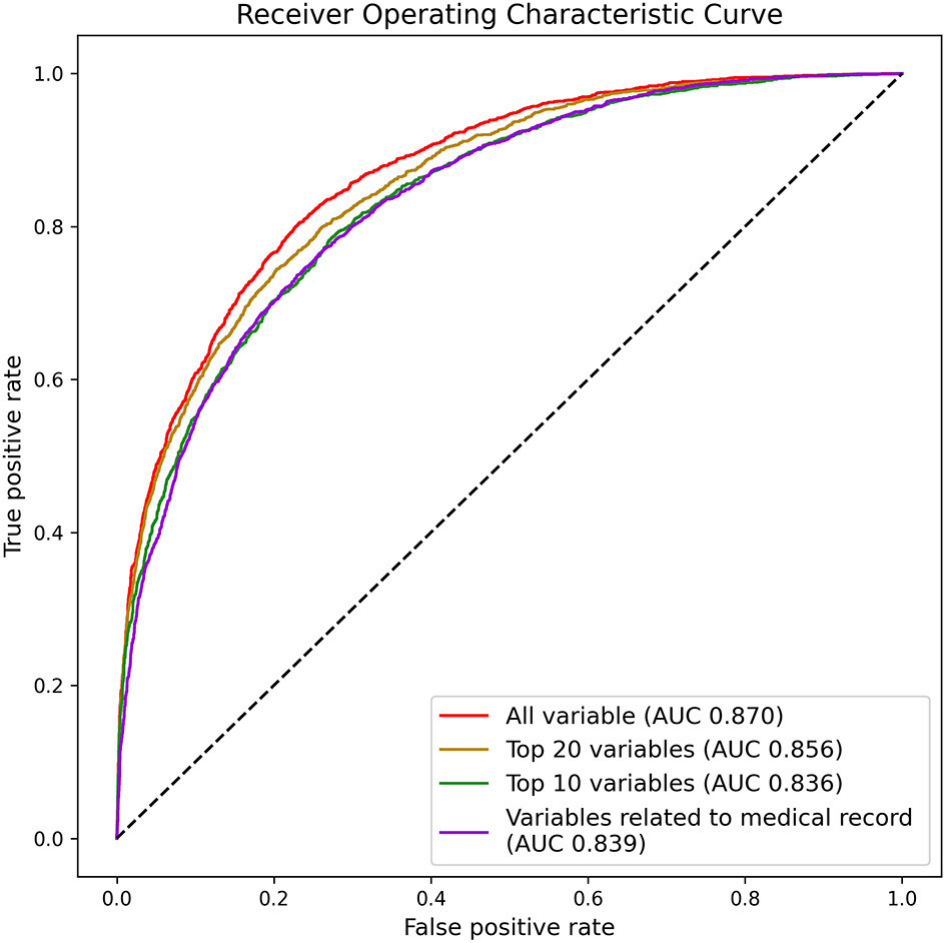
Area under the receiver operating characteristic curve for predicting hazardous drinkers by top ranked variables or by variables related to medical record in K-NHANES.

In the process of predicting hazardous drinkers, we trained the deep learning model using only specific variables (Figure 3). First, deep learning was trained with the top 20 variables based on findings about contribution of variables. Accordingly, the performance reached an AUC of 0.856. As a result of learned with the top 10 variables, the AUC was 0.836. Based on these results, it could be seen that the rate detection was preserved even when learning was performed on only 10 variables. In addition, we trained the model using only variables that could be extracted from individual medical records to see the clinical applicability of the model. Among 325 variables, 156 were selected as variables that could be extracted from personal medical records. These were laboratory findings such as AST and ALT, body measurements such as weight and height, and the presence or absence of various comorbid diseases. These variables related to medical records are listed in Supplementary Table 1. As a result of learning based on these 156 variables, an AUC of 0.839 was obtained, indicating that the performance was relatively preserved.

### Predicting the Severity of Alcohol-Related Problems From K-NHANES Dataset

**Figure 4:**
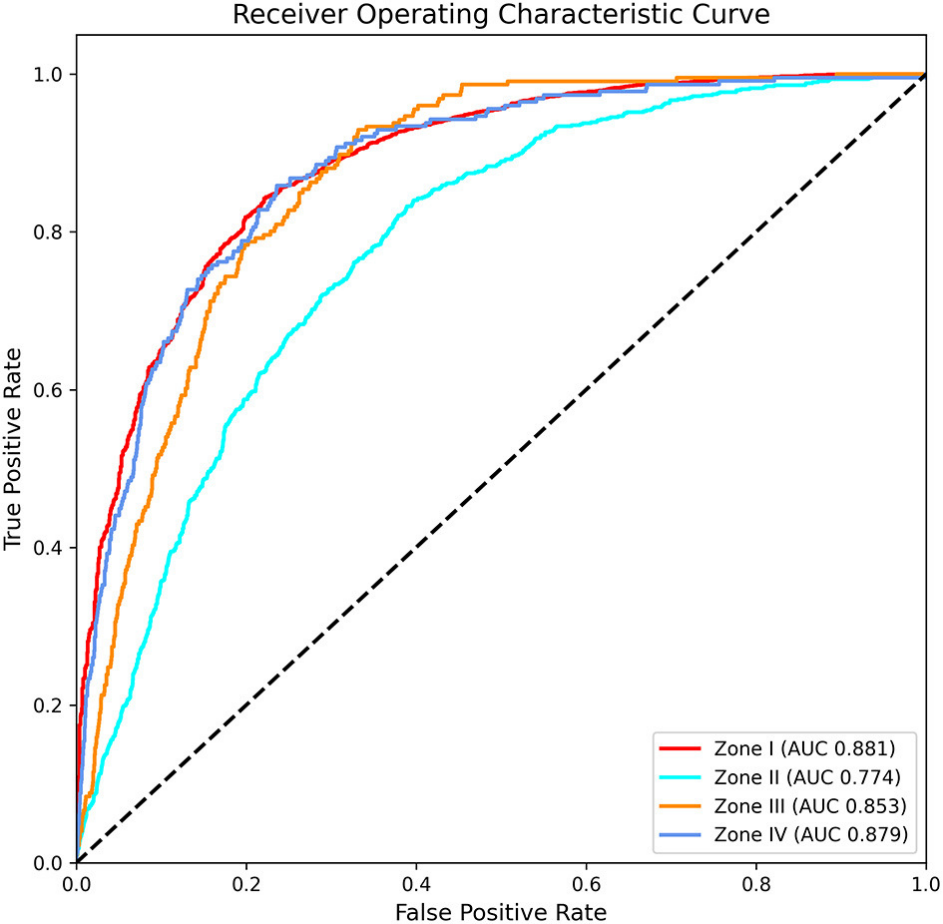
Area under the receiver operating characteristic curve for predicting the severity of alcohol-related problems in K-NHANES.

To predict the degree of individuals' problematic drinking behavior, we used a deep learning model that best predicted hazardous drinkers among various machine learning algorithms. A total of 45,672 individuals and 392 variables were used to train this model. Results are summarized in Figure 4. The model classified Zone I, Zone II, Zone III, and Zone IV with AUCs of 0.881, 0.774, 0.853, and 0.879, respectively (Accuracy: 0.763; Precision: 0.461; Recall: 0.376; F1-score: 0.386). It could be seen that the accuracy of the model for predicting the severity of alcohol-related problems was relatively preserved.

## Discussion

Through this study, we found that deep learning techniques could effectively predict hazardous drinking groups in K-NHANES, a large-scale general population dataset of South Korea. Deep learning showed significantly higher performance than conventional machine learning such as support vector machine and logistic regression. Furthermore, we were able to effectively classify individuals into Zone I, Zone II, Zone III, and Zone IV according to the severity of alcohol-related problems. These predictions were based on the definition of the WHO guideline for hazardous drinkers and severity of alcohol-related problems (Zones I–V). To the best of our knowledge, this is the first study to develop deep learning models for predicting hazardous drinkers and severity of alcohol-related problems (Zones I–V).

Previous studies have effectively predicted psychiatric diseases such as depression using large scale data. Similar to our study, one research team has predicted patients of depression in the general population with an AUC of 0.89 using a deep learning technique based on K-NHANES (16). In addition, another group has predicted the response of patients with major depressive disorder to selective serotonin reuptake inhibitors with an AUC of 0.82 by deep learning (40). One study has created a deep learning model for predicting the severity of major depressive disorder with a total of five steps with AUC ranging from 0.63 to 0.76 (41). These findings imply that deep learning is effective in predicting the diagnosis and severity of psychiatric disorders. Findings of our study were encouraging because our results were similar to or better than these previous studies.

In the process of predicting hazardous drinker, the contribution of each variable was calculated based on the value of AUC decreased after excluding that variable. As a result, energy intake, carbohydrate intake, fat intake, protein intake, and dietary intake were ranked high (ranked the 1st, 2nd, 3rd, 7th, and 11th, respectively, Table 1). The *T*-test also showed that the average intake of each of these variables was significantly higher in hazardous drinkers than other participants (*t* = 53.1, *p* < 0.001; *t* =12.0, *p* < 0.001; *t* = 36.3, *p* < 0.001; *t* = 42.3, *p* < 0.001; *t* = 47.6, *p* < 0.001, respectively). This is consistent with results of previous studies showing a strong evidence that hazardous drinkers have a significantly higher overall energy intake (42, 43). It may be due to high calories of alcohol itself in part. One study showed that an average of about 60% of a hazardous drinker's energy intake comes from alcohol (44). In addition, hazardous drinkers are known to show significantly higher carbohydrate, protein, and fat intake (44). The same trend was observed in the dataset used in our study.

There are additional explanations for why energy and macronutrient intake have become major factors in predicting hazardous drinkers. Alcohol use disorder is often comorbid with other psychiatric and personality disorders, especially with eating disorders (45). Several studies have shown that the lifetime comorbidity of eating disorder and alcohol use disorder in women was 15–32%, which is significantly higher than the general population (46–49). To the specific, bulimia nervosa and the bulimic subtype of anorexia nervosa were more common than restricting anorexia nervosa in alcoholism (47). Another study also reported that 20% of patients with alcohol use disorder showed binge eating, and 12% of the patients had some form of inappropriate compensatory behaviors related to weight gain (50, 51). Studies of the psychopathology of this relationship reported that impulsivity had a significant impact on the correlation between problematic eating behavior and alcohol intake (52, 53). In addition, alcohol use disorders and eating disorders have similar neurobiological backgrounds. Both disorders are associated with dysfunctional opioid and dopaminergic pathways (54). Furthermore, they show reduced activity in the brain areas associated with self-control such as the orbito-frontal and pre-frontal cortex areas (54). Therefore, because of the strong association between indiscriminate alcohol consumption and inappropriate eating habits, energy and macronutrient intake may be major factors in predicting hazardous drinkers.

As another major predictor, HDL-cholesterol was ranked 5th in the contribution ranking. The HDL level of hazardous drinkers was significantly higher in the dataset (*t*-test: *t* = 16.1, *p* < 0.001). This is consistent with previous studies showing that alcohol consumption can increase transport rates of HDL apolipoproteins ApoA-I and ApoA-II (55, 56). Triglyceride was ranked the 9th as a contribution factor in the present study. Its level was significantly higher in hazardous drinkers. Such higher levels in hazardous drinkers might be due to a decrease in the breakdown of chylomicrons and VLDL remnants caused by the inhibitory effect of alcohol (*t* = 32.0, *p* < 0.001) (57). Waist circumstance and BMI were ranked 14th and 19th as contributing factors in the present study. They were significantly higher in hazardous drinkers (*t* = 28.9, *p* < 0.001; *t* = 13.8, *p* < 0.001, respectively). Alcohol consumption has known to be closely related to weight gain. It especially increases waist circumference (58, 59). Thus, waist circumstance and BMI seem to be strong predictors for hazardous drinkers.

In our results, the ranking of numerical variables was generally higher. Of a total of 324 variables used in the analysis, only 88 (27.2%) were numerical variables. Among the top 20 variables, only three were categorical variables whereas 17 were numerical variables. This might be because numeric variables provided more detailed clinical information about patients. On the other hand, if the purpose of this study was to find factors contributing to the prediction of Zone IV group, a more serious group than hazardous drinkers, results would be different. For example, despite the fact that AST and ALT ranked the 10th and the 11th as contributors for classifying hazardous drinkers, they might result in a greater contribution in predicting the Zone IV group considering liver enzymes are known to deteriorate markedly in a more severe group (60).

The AUC was 0.856 for predicting hazardous drinkers with top 20 variables and 0.836 for such prediction with top 10 variables, suggesting that hazardous drinkers could be effectively predicted with only a few variables. In addition, of a total of 325 variables, when 156 variables that could be automatically extracted from hospital medical records were used, a high AUC value of 0.839 was obtained. Therefore, the deep learning model can be applied to a hospital system so that patients who visit departments other than psychiatry can be automatically consulted if they are hazardous drinkers.

In clinical settings, it is practically difficult to screen all patients for alcohol use. One study has used AUDIT-C for all patients visiting the emergency room for 1 year and found that only 65% of patients are screened (61). It was found that 25% of patients did not undergo such screening because their medical staff forgot to ask questions about alcohol and 8.8% of patients struggled with questions or refused to cooperate. However, if the deep learning model of this study could be embedded in a hospital system, it could be used to screen all patients based on their medical records, which relieves the burden of medical staffs and avoids the problems with patients' refusal. Variables extracted from hospital medical records were sufficient enough to adequately predict hazardous drinkers in this model. Therefore, even without additional information, such as nutritional status, it is possible to predict whether a patient is a hazardous drinker based on the data already collected in the hospital. In other words, automated screening through deep learning can overcome limitations of medical resources and factors associated with medical staff and patients that may appear in the clinical field.

In addition, the deep learning algorithm established in this study might be used to predict the prevalence of hazardous drinkers in a specific group. In the absence of a mental health survey in a specific region, the regional prevalence of hazardous drinkers could be estimated through a trained deep learning model of this study. Even in an environment where health surveys could not be conducted every year, it is possible to infer how the trend of prevalence of hazardous drinkers changes over time through the trained deep learning model.

Our results showed that the severity of an individual's alcohol-related problems could be predicted successfully. In a clinical setting, there might be situations where there is insufficient time to query the entire AUDIT questions, or where the patients refuse to report or reduce their symptoms due to the stigma of alcohol-related disease. In such a situation, a trained deep learning algorithm of this study would be helpful in assessing the severity of alcohol-related problems in patients. Patients with alcohol use disorders can receive outpatient medication, intensive behavioral programs, or inpatient treatment depending on their severity (62). Using the deep learning algorithm, the severity of alcohol problems in patients could be analyzed more efficiently, thus facilitating the treatment plan, period and the prediction of prognosis.

This study has several limitations. First, this study was conducted on the general population of Korea. Thus, different results might be obtained for a population from different countries or with different cultures. Although AUDIT was developed as an international instrument, it should be applied in consideration of each country's culture, society, and environment (12). Second, since our datasets were based on cross-sectional data, it was difficult to judge the future prognosis of patients. Although we predicted hazardous drinkers and the extent of alcohol-related problems at the time of investigation, longitudinal data would be needed to determine their prognosis. Third, in the process of calculating the contribution of each variable in this study, the greater the correlation between one variable with other variables, the more likely that its contribution was underestimated. For example, if a variable was closely related to another variable, removing one of them might not significantly affect the model performance. Thus, the contribution of that variable might have been undervalued.

In conclusion, the current study found that deep learning could successfully predict hazardous drinkers and classify individuals according to the severity of their alcohol-related problems. In addition, it was possible to find out which factors were more important in the process. This means that deep learning can bring about a great increase in the efficiency of diagnosis and treatment of alcohol-related diseases. However, this study was based on cross-sectional data. Additional studies on the prediction of treatment response and long-term prognosis of patients based on longitudinal data are needed in the future.

## Data Availability Statement

Publicly available datasets were analyzed in this study. This data can be found here: https://knhanes.kdca.go.kr.

## Ethics Statement

The studies involving human participants were reviewed and approved by the Institutional Review Board of the Ethics Committee of St. Vincent's Hospital at The Catholic University of Korea (VC21ZISI0042). The patients/participants provided their written informed consent to participate in this study.

## Author Contributions

S-YK: conceptualization, investigation, data curation, formal analysis, and writing – original draft. TP: conceptualization, investigation, formal analysis, and writing – original draft. KK: investigation, formal analysis, and supervision. JO: formal analysis, writing – review and editing, and supervision. YP: investigation and formal analysis. D-JK: conceptualization, investigation, data curation, writing – review and editing, and project administration. All authors commented and supervised on the manuscript and approved the final version of the manuscript.

## Conflict of Interest

The authors declare that the research was conducted in the absence of any commercial or financial relationships that could be construed as a potential conflict of interest.
