# Supplementary material for: A Deep Learning Algorithm to Predict Hazardous Drinkers and the Severity of Alcohol-Related Problems Using K-NHANES
Source: Front Psychiatry. 2021 Jul 9;12:684406. doi: 10.3389/fpsyt.2021.684406 (PMC8299053; doi:10.3389/fpsyt.2021.684406)
Supplement: Supplementary file 1 [file Data_Sheet_1.docx]

**Supplementary Methods**

Managing the entire AUDIT questionnaire consisting of 10 questions is often difficult during overloaded outpatient care or emergency care. In fact, there are many abbreviated versions of AUDIT, including AUDIT-QF (only contains Questions 1 and 2 of AUDIT), AUDIT-C (Questions 1, 2, 3), and AUDIT-4 (Questions 1, 2, 3, 10). These abbreviated versions are widely used in various situations.

Not all AUDIT questions have always been included in K-NHANES since the start of K-NHANES. K-NHANES IV, V, and a part of VI (2013, 2015) included all AUDIT questions in the survey. However, K-NHANES VII and a part of VI (2014) used AUDIT-4 (Questions 1, 2, 3, 10), a simplified version of AUDIT.

The WHO guideline for AUDIT suggests that eight or more points could be the cut-off value of hazardous drinkers. At the same time, the selection of cut-off value should be adjusted based on national and cultural standards. It also suggests that setting the cut-off point for men over 65 years and women of all ages to 7 points would increase the sensitivity for these groups because effects of alcohol vary depending on the average weight and metabolism.

A great deal of research has been done on the abbreviated version of AUDIT and a variety of cut-off values have been reported. In a precedent study (listed in the 25th of the References), the cut-off value for Korean hazardous drinkers was obtained from abbreviated versions of AUDIT based on K-NHANES data. According to that study through subgroup analysis, the most appropriate cut-off value for AUDIT-4 was 7 points for men and 5 points for women and the elderly.

In other words, to identify hazardous drinkers from datasets, the cut-off value for K-NHANES IV, V, a part of VI (2013, 2015) which introduced full version of AUDIT was 8 points for males and 7 points for females and older adults, while the cut-off value for VII and a part of K-NHANES VI (2014) which introduced AUDIT-4 was 7 points for males and 5 points for females and older adults.

According to WHO guidelines, if a full version of AUDIT is performed, the alcohol risk level could be assessed into four stages: Zone I, Zone II, Zone III, and Zone IV. Zone I corresponds to an AUDIT score of 0 to 7, meaning abstinence or low-risk drinking. The second tier, Zone II, includes alcohol consumption in excess of low-risk norms. It is usually indicated when the AUDIT score is 8 to 15. Short-term intervention includes simple counseling. Patient training is the most appropriate measure for such patients. The third level, Zone III, corresponds to an AUDIT score of 16 to 19. Patients in this range need to be managed by a combination of simple advice, brief counseling, and continued monitoring. The fourth level, Zone IV, is usually defined by an AUDIT score ≥ 20. These patients are advised to consult a specialist for diagnosis and treatment. Continued studies on alcohol dependence have shown that the higher the AUDIT score, the higher the severity of alcohol-related problems.

To establish a deep learning model that could predict the severity of alcohol-related problems, we needed datasets that surveyed all AUDIT questions, not abbreviated versions of AUDIT. K-NHANES VII and a part of VI (2014) asked four of 10 AUDIT questions. Thus, they could not be used as datasets for the machine learning model to classify the severity. Instead, K-NHANES IV, V, and a part of VI (2013, 2015) were used to categorize the severity as these surveyed all AUDIT questions.

Supplementary Table 1. Contribution ranking of all variables used to predict hazardous drinkers in K-NHANES and variables that could be extracted from medical records.

| Contribution ranking | Variable code | Variable description | AUC obtained by  excluding this variable | | Variables related to medical record |
| --- | --- | --- | --- | --- | --- |
| 1 | N_EN | Energy intake (Kcal) | | 0.6179801 |  |
| 2 | N_CHO | Carbohydrate intake (g) | | 0.7757575 |  |
| 3 | N_FAT | Fat intake (g) | | 0.8372522 |  |
| 4 | age | Age | | 0.8387202 | ○ |
| 5 | HE_HDL_st2 | HDL-cholesterol | | 0.8564337 | ○ |
| 6 | sex | Sex | | 0.856489 | ○ |
| 7 | N_PROT | Protein intake (g) | | 0.8607539 |  |
| 8 | HE_RBC | Red blood cells | | 0.8614541 | ○ |
| 9 | HE_TG | Triglyceride | | 0.8624155 | ○ |
| 10 | HE_ast | Aspartate aminotransferase | | 0.8633011 | ○ |
| 11 | HE_alt | Alanine aminotransferase | | 0.8653823 | ○ |
| 12 | HE_HB | Hemoglobin | | 0.8653889 | ○ |
| 13 | BS1_1 | (Adult) Lifetime smoking | | 0.8667786 | ○ |
| 14 | HE_wc | Waist circumference | | 0.8674219 |  |
| 15 | HE_chol | Total cholesterol | | 0.8681976 | ○ |
| 16 | BS3_1 | Current smoking status | | 0.8683784 | ○ |
| 17 | N_INTK | Dietary intake (g) | | 0.8683819 |  |
| 18 | HE_HCT | Hematocrit | | 0.8689445 | ○ |
| 19 | HE_BMI | Body mass index | | 0.8692696 | ○ |
| 20 | edu | Education level reclassification code | | 0.8693084 |  |
| 21 | HE_glu | Glucose | | 0.8693738 | ○ |
| 22 | HE_crea | Blood creatinine | | 0.8693999 | ○ |
| 23 | EC_lgw_2 | The job with the longest time  : Standard job classification | | 0.8694157 |  |
| 24 | BS5 | Nicotine dependence | | 0.8694797 | ○ |
| 25 | BS8_2 | Exposure to secondhand smoke in the workplace | | 0.8695014 |  |
| 26 | HE_prg | Pregnancy | | 0.8695605 | ○ |
| 27 | HE_mens | Menstruation | | 0.8695932 |  |
| 28 | N_WAT_C | Water intake (cup) | | 0.869602 |  |
| 29 | HE_WBC | White blood cells | | 0.8696495 | ○ |
| 30 | N_NA | Sodium Intake (mg) | | 0.8696716 |  |
| 31 | sm_presnt | Current smoking rate | | 0.8696756 |  |
| 32 | marri_2 | marital status | | 0.8696832 |  |
| 33 | BS2_1 | Age to start smoking | | 0.8696925 |  |
| 34 | BO3_05 | Whether to use weight loss drugs  (not prescribed drugs) | | 0.8696988 |  |
| 35 | HE_BUN | Blood urea nitrogen | | 0.8697065 | ○ |
| 36 | BH9_11 | Influenza (flu) vaccination | | 0.8697143 |  |
| 37 | npins | Whether to join private health insurance | | 0.8697216 |  |
| 38 | BE5_1 | Number of days of muscle power exercise per week | | 0.869752 |  |
| 39 | N_WATER | Water intake (g) | | 0.869771 |  |
| 40 | EC1_2 | Reasons for Unemployment | | 0.8697796 |  |
| 41 | BP6_10 | Whether you have ever thought of suicide in a year | | 0.8697851 |  |
| 42 | EC_wht_5 | Pattern of working hours | | 0.8697862 |  |
| 43 | L_OUT_FQ | Number of times to eat out | | 0.8697914 |  |
| 44 | HE_Uro | Urobilinogen | | 0.86981 | ○ |
| 45 | marri_1 | Whether to be married for life | | 0.8698133 |  |
| 46 | BS9_2 | Exposure to secondhand smoke in the home | | 0.8698179 |  |
| 47 | HE_PLS | Pulse rate (15 sec) | | 0.8698198 |  |
| 48 | BS5_1 | Smoking cessation plan within 1 month | | 0.8698224 |  |
| 49 | BS3_2 | Average daily smoking amount | | 0.8698288 |  |
| 50 | HE_ht | Height | | 0.8698376 | ○ |
| 51 | HE_hCHOL | Hypercholesterolemia | | 0.8698387 | ○ |
| 52 | BH1 | Health checkup status | | 0.8698416 |  |
| 53 | educ | Education level | | 0.8698436 |  |
| 54 | HE_Usg | Urine specific gravity | | 0.8698598 | ○ |
| 55 | N_DIET | Diet therapy | | 0.86986 |  |
| 56 | BM7 | Chewing problem | | 0.8698646 |  |
| 57 | BP5 | Whether or not you feel depressed for  more than 2 consecutive weeks | | 0.8698669 |  |
| 58 | HE_Bplt | Platelet | | 0.8698689 | ○ |
| 59 | mh_stress | Peception of individual stress | | 0.8698692 |  |
| 60 | N_VITC | Vitamin C intake (mg) | | 0.8698752 |  |
| 61 | HE_hp | Presence of hypertension | | 0.869877 | ○ |
| 62 | sc_seatblt2 | Seat belt use rate when riding on passenger seat | | 0.8698809 |  |
| 63 | BO1 | Subjective recognition of body shape | | 0.8698822 |  |
| 64 | BO1_1 | Whether the weight change for 1 year | | 0.8698869 |  |
| 65 | occp | Occupational Reclassification and Unemployment /Inactivity Demographic Status Code | | 0.869887 |  |
| 66 | ho_incm5 | Income quintile (household) | | 0.8698885 |  |
| 67 | BO3_04 | Whether to use weight loss drugs  (prescribed drugs) | | 0.8698901 |  |
| 68 | BP1 | The degree of stress in daily life | | 0.8698908 |  |
| 69 | HE_obe | Obesity | | 0.8698937 | ○ |
| 70 | graduat | Education level - graduation status | | 0.8698957 |  |
| 71 | LF_SAFE | The eating habits | | 0.8698999 |  |
| 72 | LK_LB_EF | Whether you are affected by the nutritional label of the food | | 0.8699003 |  |
| 73 | HE_Uph | Urine pH | | 0.8699024 | ○ |
| 74 | BS6_2_1 | Past smoking period (year, currently no smoking) | | 0.8699027 |  |
| 75 | BO2_1 | Weight control in past year | | 0.8699045 |  |
| 76 | BS3_3 | Number of smoking days in the last month | | 0.869906 |  |
| 77 | HE_dbp2 | Secondary diastolic blood pressure | | 0.8699079 |  |
| 78 | BS5_30 | Never tried to quit smoking | | 0.8699109 |  |
| 79 | DE1_ag | When to diagnose diabetes | | 0.869915 | ○ |
| 80 | BS5_28 | Method used for quitting smoking  : Non-smoking call center | | 0.8699172 |  |
| 81 | HE_HBsAg | Hepatitis B surface antigen | | 0.8699179 | ○ |
| 82 | BS5_29 | Method used for quitting smoking : Other way | | 0.8699183 |  |
| 83 | EC_occp | Standard occupational classification : Large category code | | 0.8699196 |  |
| 84 | BM2_3 | Use of interdental toothbrush | | 0.869921 |  |
| 85 | BM1_8 | Brushing time : before going to sleep | | 0.8699229 |  |
| 86 | N_NIAC | Niacin intake (mg) | | 0.8699232 |  |
| 87 | EC_lgw_4 | The job with the longest time : employment status | | 0.8699242 |  |
| 88 | BO1_2 | Weight loss per year | | 0.8699247 |  |
| 89 | LQ_5EQL | EuroQoL : anxiety or depression | | 0.8699288 |  |
| 90 | N_FE | Iron intake (mg) | | 0.8699289 |  |
| 91 | DC4_dg | Diagnosis of breast cancer | | 0.8699312 | ○ |
| 92 | HE_dbp | Final diastolic blood pressure (2nd and 3rd mean) | | 0.8699321 | ○ |
| 93 | BM1_6 | Brushing time : after dinner | | 0.8699328 |  |
| 94 | BM1_2 | Brushing time: after breakfast | | 0.8699339 |  |
| 95 | BS6_2 | Past smoking period (Converted to month, currently no smoking) | | 0.8699346 |  |
| 96 | N_RETIN | Retinol intake (μg) | | 0.869935 |  |
| 97 | EC_wh | Current job :  full-time or part-time | | 0.8699358 |  |
| 98 | HE_sbp | Final systolic blood pressure (2nd and 3rd mean) | | 0.8699365 | ○ |
| 99 | DF2_dg | Diagnosis of depression | | 0.8699395 | ○ |
| 100 | LK_LB_IT | Nutrients of interest in nutrition labeling | | 0.8699405 |  |
| 101 | cfam | Number of people in a household | | 0.8699409 |  |
| 102 | genertn | Household Generation Code | | 0.8699414 |  |
| 103 | DE1_dg | Diagnosis of diabetes | | 0.8699418 | ○ |
| 104 | BS5_26 | Method used for quitting smoking :  Public Health Center Smoking Clinic | | 0.8699458 |  |
| 105 | DI2_ag | When to diagnose dyslipidemia | | 0.8699458 | ○ |
| 106 | DI1_2 | Blood pressure medication | | 0.8699458 | ○ |
| 107 | DE1_31 | Glucose control treatment : insulin injection | | 0.8699462 | ○ |
| 108 | fam_rela | Relationship with householder | | 0.8699473 |  |
| 109 | EC_stt_2 | Current job :  detailed employment status | | 0.8699477 |  |
| 110 | ainc_unit1 | Household Income (Open Type): Income Unit | | 0.8699484 |  |
| 111 | BA2_12 | Whether to wear a seat belt while driving | | 0.8699489 |  |
| 112 | LQ4_01 | Reasons for restriction of activity : fracture, joint injury | | 0.8699498 |  |
| 113 | DE1_pt | Whether to treat diabetes | | 0.8699504 | ○ |
| 114 | HE_DM | Presence of Diabetes mellitus | | 0.8699515 | ○ |
| 115 | BH2_61 | Cancer screening for 2 years | | 0.869952 |  |
| 116 | HE_Uket | Urine ketone | | 0.8699525 | ○ |
| 117 | house | Home ownership | | 0.869953 |  |
| 118 | BP6_31 | Whether you have attempted suicide for a year | | 0.8699533 |  |
| 119 | DC11_pr | Whether you are currently suffering  from other cancer 1 | | 0.8699534 | ○ |
| 120 | D_1_1 | Subjective health status | | 0.8699538 |  |
| 121 | HE_hepaB | Hepatitis B surface antigen positive | | 0.8699543 | ○ |
| 122 | BA2_13 | Whether to wear a seat belt  in the front seat of the car | | 0.8699551 |  |
| 123 | town_t | Type of town, the house belongs to | | 0.8699552 |  |
| 124 | N_PHOS | Phosphorus intake (mg) | | 0.8699556 |  |
| 125 | DC11_dg | Diagnosis of other cancer 1 | | 0.8699558 | ○ |
| 126 | BM2_4 | Supplies: electric toothbrush | | 0.8699563 |  |
| 127 | HE_fst | Fasting time | | 0.8699567 |  |
| 128 | BO3_02 | Weight control method : fasting (more than 24 hours) | | 0.8699568 |  |
| 129 | DE1_32 | Glucose control treatment : diabetes medication | | 0.8699571 | ○ |
| 130 | DC4_pt | Whether to treat breast cancer | | 0.8699574 | ○ |
| 131 | DE2_dg | Diagnosis of thyroid disease | | 0.8699578 | ○ |
| 132 | DE1_34 | Glucose control treatment : other | | 0.8699579 | ○ |
| 133 | DM3_pt | Whether to treat rheumatoid arthritis | | 0.8699586 | ○ |
| 134 | DC2_dg | Diagnosis of hepatic carcinoma | | 0.8699589 | ○ |
| 135 | incm | Quartile of income (personal) | | 0.8699591 |  |
| 136 | DI2_dg | Diagnosis of dyslipidemia | | 0.8699592 | ○ |
| 137 | DM2_dg | Diagnosis of osteoarthritis | | 0.86996 | ○ |
| 138 | LQ4_05 | Reasons for restriction of activity : respiratory problems, lung disease, asthma | | 0.8699606 |  |
| 139 | DC2_ag | When to diagnose hepatic carcinoma | | 0.8699608 | ○ |
| 140 | DJ4_dg | Diagnosis of asthma | | 0.869961 | ○ |
| 141 | BO3_09 | Weight control method : one Food Diet | | 0.8699625 |  |
| 142 | DI4_pt | Whether to treat myocardial infarction or angina | | 0.8699627 | ○ |
| 143 | N_CA | Calcium intake (mg) | | 0.8699628 |  |
| 144 | DE2_ag | When to diagnose thyroid disease | | 0.869964 | ○ |
| 145 | BS5_21 | Method used for quitting smoking : by own will | | 0.8699641 |  |
| 146 | BM8 | Speaking problems | | 0.8699644 |  |
| 147 | DC4_pr | Whether you are currently suffering  from breast cancer | | 0.869965 | ○ |
| 148 | DC1_pt | Whether to treat gastric cancer | | 0.8699656 | ○ |
| 149 | DI1_ag | When to diagnose high blood pressure | | 0.8699659 | ○ |
| 150 | DL1_pt | Whether to treat atopic dermatitis | | 0.8699659 | ○ |
| 151 | DI5_pt | Whether to treat myocardial infarction | | 0.8699663 | ○ |
| 152 | DK8_pt | Whether to treat hepatitis B | | 0.8699663 | ○ |
| 153 | HE_Unitr | Urine nitrite | | 0.869967 | ○ |
| 154 | ainc_1 | Household Income (Open Type): Income | | 0.8699682 |  |
| 155 | LQ4_08 | Reasons for restriction of activity : hypertension | | 0.8699683 |  |
| 156 | DK4_pr | Whether you are currently suffering  from liver cirrhosis | | 0.8699684 | ○ |
| 157 | LQ4_23 | Reasons for restriction of activity : other | | 0.8699691 |  |
| 158 | DL1_ag | When to diagnose atopic dermatitis | | 0.8699703 | ○ |
| 159 | EC_lgw_5 | The job with the longest time : detailed employment status | | 0.869971 |  |
| 160 | HE_Uglu | Cadmium | | 0.8699713 | ○ |
| 161 | DI5_dg | Diagnosis of myocardial infarction | | 0.8699714 | ○ |
| 162 | LQ4_12 | Reasons for restriction of activity : vision problems | | 0.8699716 |  |
| 163 | DI4_dg | Diagnosis of myocardial infarction or angina | | 0.8699717 | ○ |
| 164 | DC11_pt | Whether to treat other cancer 1 | | 0.8699718 | ○ |
| 165 | DE1_3 | Whether to get glucose control treatment | | 0.8699718 | ○ |
| 166 | DC1_pr | Whether you are currently suffering  from gastric cancer | | 0.869972 | ○ |
| 167 | DE1_pr | Whether you are currently suffering  from diabetes | | 0.869972 | ○ |
| 168 | DC5_ag | When to diagnose cervical cancer | | 0.8699722 | ○ |
| 169 | DI2_pt | Whether to treat dyslipidemia | | 0.8699722 | ○ |
| 170 | HE_HPdg | Diagnosis of hypertension | | 0.8699722 | ○ |
| 171 | HE_Upro | Urine protein | | 0.8699725 | ○ |
| 172 | DE2_pt | Whether to treat thyroid disease | | 0.8699729 | ○ |
| 173 | DI6_pt | Whether to treat angina | | 0.8699729 | ○ |
| 174 | BO1_3 | Weight gain per year | | 0.8699737 |  |
| 175 | DI2_pr | Whether you are currently suffering  from dyslipidemia | | 0.8699737 | ○ |
| 176 | LQ4_00 | Activity restriction | | 0.8699739 |  |
| 177 | allownc | Basic living status | | 0.8699739 |  |
| 178 | EC_pedu_1 | Childhood Environment: father's Education Level | | 0.8699739 |  |
| 179 | HE_DMdr | Whether diabetes medicine is taken  on the day of the examination | | 0.8699743 |  |
| 180 | N_CAROT | Carotene intake (mg) | | 0.8699743 |  |
| 181 | HE_DMdg | Diagnosis of Diabetes mellitus | | 0.8699745 | ○ |
| 182 | BM1_5 | Brushing time: before dinner | | 0.8699746 |  |
| 183 | EC1_1 | Status of economic activity | | 0.869975 |  |
| 184 | DC6_ag | When to diagnose lung cancer | | 0.8699759 | ○ |
| 185 | DK9_ag | When to diagnose hepatitis C | | 0.869976 | ○ |
| 186 | DK4_pt | Whether to treat liver cirrhosis | | 0.8699763 | ○ |
| 187 | DC1_ag | When to diagnose gastric cancer | | 0.8699764 | ○ |
| 188 | DJ4_ag | When to diagnose asthma | | 0.8699766 | ○ |
| 189 | ainc | Monthly average household income | | 0.8699768 |  |
| 190 | apt_t | Housing Type | | 0.8699774 |  |
| 191 | LQ4_14 | Reasons for restriction of activity : dementia | | 0.8699776 |  |
| 192 | DC3_dg | Diagnosis of colorectal cancer | | 0.8699777 | ○ |
| 193 | DK8_dg | Diagnosis of hepatitis B | | 0.8699783 | ○ |
| 194 | BM1_7 | Brush Time: After Snack | | 0.8699784 |  |
| 195 | BO3_03 | Weight control method : reduced meals | | 0.8699786 |  |
| 196 | LQ4_21 | Reasons for restriction of activity : obesity | | 0.8699788 |  |
| 197 | LQ4_07 | Reasons for restriction of activity : diabetes | | 0.8699789 |  |
| 198 | HE_Ubil | Urine bilirubin | | 0.8699793 | ○ |
| 199 | LQ4_16 | Reasons for restriction of activity : mental retardation | | 0.8699796 |  |
| 200 | DC2_pt | Whether to treat hepatic carcinoma | | 0.8699798 | ○ |
| 201 | DI3_ag | When to diagnose stroke | | 0.8699803 | ○ |
| 202 | BO3_12 | Weight control method : chinese medicine | | 0.8699806 |  |
| 203 | DI3_pt | Whether to treat stroke | | 0.8699809 | ○ |
| 204 | DF2_ag | When to diagnose depression | | 0.8699809 | ○ |
| 205 | DN1_dg | Diagnosis of kidney failure | | 0.869981 | ○ |
| 206 | DI3_pr | Whether you are currently suffering  from stroke | | 0.8699811 | ○ |
| 207 | BO3_01 | Weight control method : exercise | | 0.8699812 |  |
| 208 | DC5_pt | Whether to treat cervical cancer | | 0.8699814 | ○ |
| 209 | N_K | Potassium intake (mg) | | 0.8699815 |  |
| 210 | DI1_dg | Diagnosis of high blood pressure | | 0.8699819 | ○ |
| 211 | DI3_dg | Diagnosis of stroke | | 0.8699823 | ○ |
| 212 | DL1_dg | Diagnosis of atopic dermatitis | | 0.8699823 | ○ |
| 213 | HE_dbp3 | Diastolic blood pressure (3rd) | | 0.8699827 |  |
| 214 | DK4_dg | Diagnosis of liver cirrhosis | | 0.8699828 | ○ |
| 215 | EC_stt_1 | Current job :  employment status | | 0.8699831 |  |
| 216 | DE1_33 | Glucose control treatment : non-drug therapy | | 0.8699832 | ○ |
| 217 | D_2_wk | Number of days of discomfort in the last 2 weeks | | 0.8699832 |  |
| 218 | LQ2_mn | Number of days of absence in the last month | | 0.8699833 |  |
| 219 | LQ4_03 | Reasons for restriction of activity : arthritis, rheumatism | | 0.8699833 |  |
| 220 | DM3_pr | Whether you are currently suffering  from rheumatoid arthritis | | 0.8699836 | ○ |
| 221 | DL1_pr | Whether you are currently suffering  from atopic dermatitis | | 0.8699837 | ○ |
| 222 | DM1_dg | Diagnosis of arthritis | | 0.869984 | ○ |
| 223 | LQ_1EQL | EuroQoL : mobility | | 0.8699846 |  |
| 224 | DC2_pr | Whether you are currently suffering  from hepatic carcinoma | | 0.8699847 | ○ |
| 225 | DM2_pt | Whether to treat osteoarthritis | | 0.8699848 | ○ |
| 226 | N_B1 | Thiamin intake (mg) | | 0.8699849 |  |
| 227 | DK9_pt | Whether to treat hepatitis C | | 0.8699852 | ○ |
| 228 | LQ4_10 | Reasons for restriction of activity : cancer | | 0.8699852 |  |
| 229 | DC3_ag | When to diagnose colorectal cancer | | 0.8699853 | ○ |
| 230 | N_DIET_WHY | Reasons for diet therapy | | 0.8699853 |  |
| 231 | DC5_dg | Diagnosis of cervical cancer | | 0.8699855 | ○ |
| 232 | HE_Ubld | Urine occult blood | | 0.8699857 | ○ |
| 233 | LQ4_06 | Reasons for restriction of activity : stroke | | 0.8699859 |  |
| 234 | DK9_dg | Diagnosis of hepatitis C | | 0.8699864 | ○ |
| 235 | LQ2_ab | Whether you have been absent  from work in the last month | | 0.8699865 |  |
| 236 | DM1_pr | Whether you are currently suffering  from arthritis | | 0.869987 | ○ |
| 237 | D_2_1 | Not feel very well in the past two weeks | | 0.8699871 |  |
| 238 | LQ4_09 | Reasons for restriction of activity : back and neck problems | | 0.8699874 |  |
| 239 | DC11_ag | When to diagnose other cancer 1 | | 0.8699874 | ○ |
| 240 | DJ2_pt | Whether to treat pulmonary tuberculosis | | 0.8699875 | ○ |
| 241 | DC3_pt | Whether to treat colorectal cancer | | 0.8699875 | ○ |
| 242 | BM1_3 | Brushing time: before lunch | | 0.8699879 |  |
| 243 | DK8_ag | When to diagnose hepatitis B | | 0.8699879 | ○ |
| 244 | LQ_2EQL | EuroQoL : self care | | 0.8699881 |  |
| 245 | DJ2_ag | When to diagnose pulmonary tuberculosis | | 0.8699883 | ○ |
| 246 | N_VA | Vitamin A (retinol equivalent) intake (μgRE) | | 0.8699884 |  |
| 247 | DC4_ag | When to diagnose breast cancer | | 0.8699886 | ○ |
| 248 | DJ2_pr | Whether you are currently suffering  from pulmonary tuberculosis | | 0.8699888 | ○ |
| 249 | BM1_1 | Brushing time: before breakfast | | 0.869989 |  |
| 250 | DI5_pr | Whether you are currently suffering  from myocardial infarction | | 0.8699891 | ○ |
| 251 | DC6_pr | Whether you are currently suffering  from lung cancer | | 0.8699893 | ○ |
| 252 | DI2_2 | Dyslipidemia medication | | 0.8699895 |  |
| 253 | DJ4_3 | Medication for asthma | | 0.8699895 | ○ |
| 254 | LQ4_13 | Reasons for restriction of activity : hearing problems | | 0.8699895 |  |
| 255 | BE3_32 | Walking duration (hours) | | 0.8699902 |  |
| 256 | DI4_pr | Whether you are currently suffering  from myocardial infarction or angina | | 0.8699905 | ○ |
| 257 | BM2_1 | Supplies: Floss | | 0.8699906 |  |
| 258 | DN1_pr | Whether you are currently suffering  from kidney failure | | 0.8699908 | ○ |
| 259 | DK4_ag | When to diagnose liver cirrhosis | | 0.8699908 | ○ |
| 260 | DI6_pr | Whether you are currently suffering  from angina | | 0.8699915 | ○ |
| 261 | DC6_pt | Whether to treat lung cancer | | 0.8699916 | ○ |
| 262 | DK8_pr | Whether you are currently suffering  from hepatitis B | | 0.8699916 | ○ |
| 263 | HE_wt | Weight | | 0.8699916 | ○ |
| 264 | HE_rPLS | Pulse regularity | | 0.8699922 |  |
| 265 | BO3_07 | Weight control method : health functional food | | 0.8699922 |  |
| 266 | DF2_pt | Whether to treat depression | | 0.8699922 | ○ |
| 267 | LQ4_11 | Reasons for restriction of activity : dental and oral diseases | | 0.8699926 |  |
| 268 | HE_nARM | Blood pressure measuring arm | | 0.8699927 |  |
| 269 | DC1_dg | Diagnosis of gastric cancer | | 0.8699929 | ○ |
| 270 | HE_sbp1 | Systolic pressure (1st) | | 0.8699932 | ○ |
| 271 | DJ2_dg | Diagnosis of pulmonary tuberculosis | | 0.8699933 | ○ |
| 272 | DN1_pt | Whether to treat kidney failure | | 0.8699934 | ○ |
| 273 | DM3_ag | When to diagnose rheumatoid arthritis | | 0.8699935 | ○ |
| 274 | EC_pedu_2 | Childhood Environment: mother's Education Level | | 0.869994 |  |
| 275 | DC3_pr | Whether you are currently suffering  from colorectal cancer | | 0.8699943 | ○ |
| 276 | DN1_ag | When to diagnose kidney failure | | 0.8699951 | ○ |
| 277 | BE3_33 | Walking duration (minutes) | | 0.8699955 |  |
| 278 | DI6_dg | Diagnosis of angina | | 0.8699957 | ○ |
| 279 | HE_anem | Anemia | | 0.8699957 | ○ |
| 280 | HE_sbp2 | Systolic pressure (2nd) | | 0.8699958 |  |
| 281 | DI3_2 | Stroke sequelae | | 0.8699959 | ○ |
| 282 | BO3_10 | Weight control method : other | | 0.869996 |  |
| 283 | LQ4_15 | Reasons for restriction of activity : depression/anxiety/emotional problems | | 0.8699961 |  |
| 284 | LQ4_04 | Reasons for restriction of activity : heart disease | | 0.8699967 |  |
| 285 | HE_dbp1 | Diastolic blood pressure (1st) | | 0.8699979 | ○ |
| 286 | DC5_pr | Whether you are currently suffering  from cervical cancer | | 0.8699985 | ○ |
| 287 | DI6_ag | When to diagnose angina | | 0.8699985 | ○ |
| 288 | DK9_pr | Whether you are currently suffering  from hepatitis C | | 0.8699988 | ○ |
| 289 | DM3_dg | Diagnosis of rheumatoid arthritis | | 0.8699994 | ○ |
| 290 | tins | Type of health insurance | | 0.8700004 | ○ |
| 291 | DJ4_pr | Whether you are currently suffering  from asthma | | 0.8700006 | ○ |
| 292 | HE_UCREA | Urine creatinine | | 0.870001 | ○ |
| 293 | BM1_4 | Brushing time: After lunch | | 0.8700016 |  |
| 294 | incm5 | Income quintile (personal) | | 0.8700016 |  |
| 295 | DE2_pr | Whether you are currently suffering  from thyroid disease | | 0.8700023 | ○ |
| 296 | DI1_pt | Whether to treat high blood pressure | | 0.8700025 | ○ |
| 297 | DC6_dg | Diagnosis of lung cancer | | 0.870003 | ○ |
| 298 | LQ4_22 | Reasons for restriction of activity : old age | | 0.870003 |  |
| 299 | DM2_ag | When to diagnose osteoarthritis | | 0.8700037 | ○ |
| 300 | LK_EDU | Nutrition education | | 0.8700046 |  |
| 301 | BP7 | Whether you have been consulted  for mental problems for 1 year | | 0.8700051 |  |
| 302 | DM1_pt | Whether to treat arthritis | | 0.870006 | ○ |
| 303 | live_t | Housing type | | 0.8700062 |  |
| 304 | LQ_3EQL | EuroQoL : usual activities | | 0.8700066 |  |
| 305 | N_B2 | Riboflavin intake (mg) | | 0.870007 |  |
| 306 | DF2_pr | Whether you are currently suffering  from depression | | 0.8700073 | ○ |
| 307 | LQ4_02 | Reasons for restriction of activity : other injury | | 0.8700081 |  |
| 308 | DI5_ag | When to diagnose myocardial infarction | | 0.8700087 | ○ |
| 309 | BS6_2_2 | Past smoking period (month, currently no smoking) | | 0.8700123 |  |
| 310 | LQ_4EQL | EuroQoL : pain or discomfort | | 0.8700124 |  |
| 311 | DJ4_pt | Whether to treat asthma | | 0.8700141 | ○ |
| 312 | BM2_5 | Supplies: other | | 0.8700146 |  |
| 313 | DI1_pr | Whether you are currently suffering  from high blood pressure | | 0.8700152 | ○ |
| 314 | BM2_2 | Products used: Oral solution | | 0.8700162 |  |
| 315 | LQ1_sb | Whether you have been sick in the last month | | 0.8700215 |  |
| 316 | HE_HPdr | Whether hypertension medication is taken  on the day of the examination | | 0.8700243 |  |
| 317 | ho_incm | Quartile of income (household) | | 0.8700245 |  |
| 318 | EQ5D | EQ5D | | 0.8700262 |  |
| 319 | DM2_pr | Whether you are currently suffering  from osteoarthritis | | 0.8700265 | ○ |
| 320 | OR1_2 | Oral examination in the last 1 year | | 0.8700287 |  |
| 321 | HE_sbp3 | Systolic pressure (3rd) | | 0.8700309 |  |
| 322 | BS6_3 | Average daily smoking amount of past smokers | | 0.8700315 |  |
| 323 | BE3_31 | Walking days per week | | 0.8700333 |  |
| 324 | LQ1_mn | Number of days of illness in the last month | | 0.870038 |  |

Supplementary Table 2. All variables used to predict the severity of alcohol-related problems.

| Variable code | Variable description |
| --- | --- |
| ainc_unit1 | Household Income (Open Type): Income Unit |
| apt_t | Housing Type |
| BA2_12 | Whether to wear a seat belt while driving |
| BA2_13 | Whether to wear a seat belt  in the front seat of the car |
| BE3_11 | Number of days of intense physical activity per week |
| BE3_21 | Number of days of moderate physical activity per week |
| BE3_31 | Walking days per week |
| BE3_32 | Walking duration (hours) |
| BE5_1 | Number of days of muscle power exercise per week |
| BE5_2 | Flexible exercise days per week |
| BH1 | Health checkup status |
| BH1_1 | Health screenings_self pay |
| BH1_2 | Health screenings_special insdustrial site |
| BH1_3 | Health screenings_health Insurance |
| BH1_6 | Health screenings_others |
| BH2_12 | When to test for stomach cancer |
| BH2_22 | When to test for breast cancer |
| BH2_32 | When to test for cervical cancer |
| BH2_52 | When to test for liver cancer |
| BH2_61 | Cancer screening for 2 years |
| BH2_62 | Cancer screening_self pay |
| BH2_63 | Cancer screening_self pay (partial) |
| BH2_64 | Cancer screening_others |
| BH9_11 | Influenza (flu) vaccination |
| BM7 | Chewing problem |
| BM8 | Speaking problems |
| BM12 | Untreated dental care |
| BM1_1 | Brushing time: before breakfast |
| BM1_2 | Brushing time: after breakfast |
| BM1_3 | Brushing time: before lunch |
| BM1_4 | Brushing time: After lunch |
| BM1_5 | Brushing time: before dinner |
| BM1_6 | Brushing time : after dinner |
| BM1_7 | Brush Time: After Snack |
| BM1_8 | Brushing time : before going to sleep |
| BM12_1 | Reasons for non-treatment of dental treatment |
| BM2_1 | Supplies: Floss |
| BM2_2 | Products used: Oral solution |
| BM2_3 | Use of interdental toothbrush |
| BM2_4 | Supplies: electric toothbrush |
| BM2_5 | Supplies: other |
| BO1 | Subjective recognition of body shape |
| BO1_1 | Whether the weight change for 1 year |
| BO1_2 | Weight loss per year |
| BO1_3 | Weight gain per year |
| BO2_1 | Weight control in past year |
| BO3_01 | Weight control method : exercise |
| BO3_02 | Weight control method : fasting (more than 24 hours) |
| BO3_03 | Weight control method : reduced meals |
| BO3_04 | Whether to use weight loss drugs  (prescribed drugs) |
| BO3_05 | Whether to use weight loss drugs  (not prescribed drugs) |
| BO3_07 | Weight control method : health functional food |
| BO3_09 | Weight control method : one Food Diet |
| BO3_10 | Weight control method : other |
| BO3_12 | Weight control method : chinese medicine |
| BP1 | The degree of stress in daily life |
| BP5 | Whether or not you feel depressed for  more than 2 consecutive weeks |
| BP7 | Whether you have been consulted  for mental problems for 1 year |
| BP6_10 | Whether you have ever thought of suicide in a year |
| BP6_31 | Whether you have attempted suicide for a year |
| BS5 | Nicotine dependence |
| BS8 | Whether to expose second-hand smoke in the workplace |
| BS1_1 | (Adult) Lifetime smoking |
| BS3_1 | Current smoking status |
| BS5_1 | Smoking cessation plan within 1 month |
| BS5_21 | Method used for quitting smoking : by own will |
| BS5_22 | Method used for quitting smoking : Nicotine replacement |
| BS5_24 | Method used for quitting smoking : Agents to reduce the desire to smoke |
| BS5_25 | Method used for quitting smoking : Physician prescription medication |
| BS5_26 | Method used for quitting smoking :  Public Health Center Smoking Clinic |
| BS5_27 | Method used for quitting smoking : Acupuncture for quitting smoking |
| BS5_28 | Method used for quitting smoking  : Non-smoking call center |
| BS5_29 | Method used for quitting smoking : Other way |
| BS5_30 | Never tried to quit smoking |
| BS6_2_2 | Past smoking period (month, currently no smoking) |
| BS8_2 | Exposure to secondhand smoke in the workplace |
| BS8_3 | Exposure time to secondhand smoke in the workplace |
| BS9_1 | Whether there are regular smokers in the home |
| BS9_2 | Exposure to secondhand smoke in the home |
| cfam | Number of people in a household |
| D_1_1 | Subjective health status |
| D_2_1 | Not feel very well in the past two weeks |
| D_2_wk | Number of days of discomfort in the last 2 weeks |
| DC1_dg | Diagnosis of gastric cancer |
| DC1_lt | Whether you have ever had gastric cancer |
| DC1_pr | Whether you are currently suffering  from gastric cancer |
| DC1_pt | Whether to treat gastric cancer |
| DC11_dg | Diagnosis of other cancer 1 |
| DC11_lt | Whether you have ever had other cancer 1 |
| DC11_pr | Whether you are currently suffering  from other cancer 1 |
| DC11_pt | Whether to treat other cancer 1 |
| DC2_dg | Diagnosis of hepatic carcinoma |
| DC2_lt | Whether you have ever had hepatic carcinoma |
| DC2_pr | Whether you are currently suffering  from hepatic carcinoma |
| DC2_pt | Whether to treat hepatic carcinoma |
| DC3_dg | Diagnosis of colorectal cancer |
| DC3_lt | Whether you have ever had colorectal cancer |
| DC3_pr | Whether you are currently suffering  from colorectal cancer |
| DC3_pt | Whether to treat colorectal cancer |
| DC4_dg | Diagnosis of breast cancer |
| DC4_lt | Whether you have ever had breast cancer |
| DC4_pr | Whether you are currently suffering  from breast cancer |
| DC4_pt | Whether to treat breast cancer |
| DC5_dg | Diagnosis of cervical cancer |
| DC5_lt | Whether you have ever had cervical cancer |
| DC5_pr | Whether you are currently suffering  from cervical cancer |
| DC5_pt | Whether to treat cervical cancer |
| DC6_dg | Diagnosis of lung cancer |
| DC6_lt | Whether you have ever had lung cancer |
| DC6_pr | Whether you are currently suffering  from lung cancer |
| DC6_pt | Whether to treat lung cancer |
| DE1_3 | Whether to get glucose control treatment |
| DE1_5 | Diabetes education |
| DE1_31 | Glucose control treatment : insulin injection |
| DE1_32 | Glucose control treatment : diabetes medication |
| DE1_33 | Glucose control treatment : non-drug therapy |
| DE1_34 | Glucose control treatment : other |
| DE1_51 | Diabetes education_hospital |
| DE1_53 | Diabetes education_public health center |
| DE1_54 | Diabetes education_others |
| DE1_dg | Diagnosis of diabetes |
| DE1_lt | Whether you have ever had diabetes |
| DE1_pr | Whether you are currently suffering  from diabetes |
| DE1_pt | Whether to treat diabetes |
| DE2_dg | Diagnosis of thyroid disease |
| DE2_lt | Whether you have ever had thyroid disease |
| DE2_pr | Whether you are currently suffering  from thyroid disease |
| DE2_pt | Whether to treat thyroid disease |
| DF2_dg | Diagnosis of depression |
| DF2_pr | Whether you are currently suffering  from depression |
| DF2_pt | Whether to treat depression |
| DI1_2 | Blood pressure medication |
| DI1_dg | Diagnosis of high blood pressure |
| DI1_lt | Whether you have ever had high blood pressure |
| DI1_pr | Whether you are currently suffering  from high blood pressure |
| DI1_pt | Whether to treat high blood pressure |
| DI2_2 | Dyslipidemia medication |
| DI2_dg | Diagnosis of dyslipidemia |
| DI2_lt | Whether you have ever had dyslipidemia |
| DI2_pr | Whether you are currently suffering  from dyslipidemia |
| DI2_pt | Whether to treat dyslipidemia |
| DI3_2 | Stroke sequelae |
| DI3_dg | Diagnosis of stroke |
| DI3_lt | Whether you have ever had stroke |
| DI3_pr | Whether you are currently suffering  from stroke |
| DI3_pt | Whether to treat stroke |
| DI4_dg | Diagnosis of myocardial infarction or angina |
| DI4_lt | Whether you have ever had angina |
| DI4_pr | Whether you are currently suffering  from myocardial infarction or angina |
| DI4_pt | Whether to treat myocardial infarction or angina |
| DI5_dg | Diagnosis of myocardial infarction |
| DI5_lt | Whether you have ever had myocardial infarction |
| DI5_pr | Whether you are currently suffering  from myocardial infarction |
| DI5_pt | Whether to treat myocardial infarction |
| DI6_dg | Diagnosis of angina |
| DI6_lt | Whether you have ever had angina |
| DI6_pr | Whether you are currently suffering  from angina |
| DI6_pt | Whether to treat angina |
| DJ2_4 | Where pulmonary tuberculosis was treated |
| DJ2_dg | Diagnosis of pulmonary tuberculosis |
| DJ2_lt | Whether you have ever had pulmonary tuberculosis |
| DJ2_op | Outpatient experience with pulmonary tuberculosis |
| DJ2_pr | Whether you are currently suffering  from pulmonary tuberculosis |
| DJ2_pt | Whether to treat pulmonary tuberculosis |
| DJ4_3 | Medication for asthma |
| DJ4_dg | Diagnosis of asthma |
| DJ4_lt | Whether you have ever had asthma |
| DJ4_pr | Whether you are currently suffering  from asthma |
| DJ4_pt | Whether to treat asthma |
| DK4_dg | Diagnosis of liver cirrhosis |
| DK4_lt | Whether you have ever had liver cirrhosis |
| DK4_pr | Whether you are currently suffering  from liver cirrhosis |
| DK4_pt | Whether to treat liver cirrhosis |
| DK8_dg | Diagnosis of hepatitis B |
| DK8_pr | Whether you are currently suffering  from hepatitis B |
| DK8_pt | Whether to treat hepatitis B |
| DK9_dg | Diagnosis of hepatitis C |
| DK9_pr | Whether you are currently suffering  from hepatitis C |
| DK9_pt | Whether to treat hepatitis C |
| DL1_dg | Diagnosis of atopic dermatitis |
| DL1_lt | Whether you have ever had atopic dermatitis |
| DL1_pr | Whether you are currently suffering  from atopic dermatitis |
| DL1_pt | Whether to treat atopic dermatitis |
| DM1_5 | Arthritis education |
| DM1_51 | Arthritis education_hotpital |
| DM1_53 | Arthritis education_public health center |
| DM1_54 | Arthritis education_oriental medicine hospital |
| DM1_dg | Diagnosis of arthritis |
| DM1_lt | Whether you have ever had arthritis |
| DM1_pr | Whether you are currently suffering  from arthritis |
| DM1_pt | Whether to treat arthritis |
| DM2_dg | Diagnosis of osteoarthritis |
| DM2_lt | Whether you have ever had osteoarthritis |
| DM2_op | Outpatient experience with osteoarthritis |
| DM2_pr | Whether you are currently suffering  from osteoarthritis |
| DM2_pt | Whether to treat osteoarthritis |
| DM3_dg | Diagnosis of rheumatoid arthritis |
| DM3_lt | Whether you have ever had rheumatoid arthritis |
| DM3_pr | Whether you are currently suffering  from rheumatoid arthritis |
| DM3_pt | Whether to treat rheumatoid arthritis |
| DN1_dg | Diagnosis of kidney failure |
| DN1_lt | Whether you have ever had kidney failure |
| DN1_pr | Whether you are currently suffering  from kidney failure |
| DN1_pt | Whether to treat kidney failure |
| EC_lgw_2 | The job with the longest time  : Standard job classification |
| EC_lgw_5 | The job with the longest time : detailed employment status |
| EC_occp | Standard occupational classification : Large category code |
| EC_pedu_1 | Childhood Environment: father's Education Level |
| EC_pedu_2 | Childhood Environment: mother's Education Level |
| EC_stt_1 | Current job :  employment status |
| EC_stt_2 | Current job :  detailed employment status |
| EC_wh | Current job :  full-time or part-time |
| EC_wht_5 | Pattern of working hours |
| EC1_1 | Status of economic activity |
| EC1_2 | Reasons for Unemployment |
| edu | Education level reclassification code |
| educ | Education level |
| genertn | Household Generation Code |
| graduat | Education level - graduation status |
| HE_anem | Anemia |
| HE_DM | Presence of Diabetes mellitus |
| HE_DMdg | Diagnosis of Diabetes mellitus |
| HE_DMdr | Whether diabetes medicine is taken  on the day of the examination |
| HE_hCHOL | Hypercholesterolemia |
| HE_hepaB | Hepatitis B surface antigen positive |
| HE_hp | Presence of hypertension |
| HE_HPdg | Diagnosis of hypertension |
| HE_HPdr | Whether hypertension medication is taken  on the day of the examination |
| HE_LHDL_st2 | Whether you have ever had low HDL cholesterol |
| HE_mens | Menstruation |
| HE_nARM | Blood pressure measuring arm |
| HE_obe | Obesity |
| HE_prg | Pregnancy |
| HE_rPLS | Pulse regularity |
| HE_Ubil | Urine bilirubin |
| HE_Ubld | Urine occult blood |
| HE_Uglu | Cadmium |
| HE_Uket | Urine ketone |
| HE_Unitr | Urine nitrite |
| HE_Upro | Urine protein |
| HE_Uro | Urobilinogen |
| ho_incm | Quartile of income (household) |
| ho_incm5 | Income quintile (household) |
| house | Home ownership |
| incm | Quartile of income (personal) |
| incm5 | Income quintile (personal) |
| L_OUT_FQ | Number of times to eat out |
| LF_CARE | Dietary support program |
| LF_SAFE | The eating habits |
| live_t | Housing type |
| LK_EDU | Nutrition education |
| LK_LB_EF | Whether you are affected by the nutritional label of the food |
| LK_LB_IT | Nutrients of interest in nutrition labeling |
| LQ_1EQL | EuroQoL : mobility |
| LQ_2EQL | EuroQoL : self care |
| LQ_3EQL | EuroQoL : usual activities |
| LQ_4EQL | EuroQoL : pain or discomfort |
| LQ_5EQL | EuroQoL : anxiety or depression |
| LQ1_sb | Whether you have been sick in the last month |
| LQ2_ab | Whether you have been absent  from work in the last month |
| LQ4_00 | Activity restriction |
| LQ4_01 | Reasons for restriction of activity : fracture, joint injury |
| LQ4_02 | Reasons for restriction of activity : other injury |
| LQ4_03 | Reasons for restriction of activity : arthritis, rheumatism |
| LQ4_04 | Reasons for restriction of activity : heart disease |
| LQ4_05 | Reasons for restriction of activity : respiratory problems, lung disease, asthma |
| LQ4_06 | Reasons for restriction of activity : stroke |
| LQ4_07 | Reasons for restriction of activity : diabetes |
| LQ4_08 | Reasons for restriction of activity : hypertension |
| LQ4_09 | Reasons for restriction of activity : back and neck problems |
| LQ4_10 | Reasons for restriction of activity : cancer |
| LQ4_11 | Reasons for restriction of activity : dental and oral diseases |
| LQ4_12 | Reasons for restriction of activity : vision problems |
| LQ4_13 | Reasons for restriction of activity : hearing problems |
| LQ4_14 | Reasons for restriction of activity : dementia |
| LQ4_15 | Reasons for restriction of activity : depression/anxiety/emotional problems |
| LQ4_16 | Reasons for restriction of activity : mental retardation |
| LQ4_21 | Reasons for restriction of activity : obesity |
| LQ4_22 | Reasons for restriction of activity : old age |
| LQ4_23 | Reasons for restriction of activity : other |
| marri_1 | Whether to be married for life |
| marri_2 | marital status |
| mh_melan | Perception of depressed mood more than 2 consecutive weeks |
| mh_stress | Perception of individual stress |
| mh_suicide | Perception of suicidal thought in recent 1 year |
| N_DIET | Diet therapy |
| N_DIET_WHY | Reasons for diet therapy |
| npins | Whether to join private health insurance |
| O_DIP | Prevalence of caries in permanent teeth |
| O_DMFIP | Experience of permanent tooth caries |
| O_DTP | Number of dentures |
| occp | Occupational Reclassification and Unemployment /Inactivity Demographic Status Code |
| OR1 | Oral health status |
| OR1_2 | Oral examination in the last 1 year |
| pa_high | Practive rate of vigorous physical activity |
| pa_mid | Practive rate of moderate physical activity |
| pa_walk | Practive rate of walking |
| sc_seatblt2 | Seat belt use rate when riding on passenger seat |
| sex | Sex |
| sm_presnt | Current smoking rate |
| town_t | Type of town, the house belongs to |
| age | Age |
| ainc | Monthly average household income |
| ainc_1 | Household Income (Open Type): Income |
| allownc | Basic living status |
| BE3_33 | Walking duration (minutes) |
| BP8 | Average sleep time per day |
| BS2_1 | Age to start smoking |
| BS3_2 | Average daily smoking amount |
| BS6_2 | Past smoking period (Converted to month, currently no smoking) |
| BS6_3 | Average daily smoking amount of past smokers |
| BS6_2_1 | Past smoking period (year, currently no smoking) |
| BS9_3 | Exposure time to secondhand smoke in the home |
| DC1_ag | When to diagnose gastric cancer |
| DC11_ag | When to diagnose other cancer 1 |
| DC2_ag | When to diagnose hepatic carcinoma |
| DC3_ag | When to diagnose colorectal cancer |
| DC4_ag | When to diagnose breast cancer |
| DC5_ag | When to diagnose cervical cancer |
| DC6_ag | When to diagnose lung cancer |
| DE1_ag | When to diagnose diabetes |
| DE2_ag | When to diagnose thyroid disease |
| DF2_ag | When to diagnose depression |
| DI1_ag | When to diagnose high blood pressure |
| DI2_ag | When to diagnose dyslipidemia |
| DI3_ag | When to diagnose stroke |
| DI5_ag | When to diagnose myocardial infarction |
| DI6_ag | When to diagnose angina |
| DJ2_ag | When to diagnose pulmonary tuberculosis |
| DJ4_ag | When to diagnose asthma |
| DK4_ag | When to diagnose liver cirrhosis |
| DK8_ag | When to diagnose hepatitis B |
| DK9_ag | When to diagnose hepatitis C |
| DL1_ag | When to diagnose atopic dermatitis |
| DM2_ag | When to diagnose osteoarthritis |
| DM3_ag | When to diagnose rheumatoid arthritis |
| DN1_ag | When to diagnose kidney failure |
| EQ5D | EQ5D |
| HE_alt | Alanine aminotransferase |
| HE_ast | Aspartate aminotransferase |
| HE_BMI | Body mass index |
| HE_Bplt | Platelet |
| HE_BUN | Blood urea nitrogen |
| HE_chol | Total cholesterol |
| HE_crea | Blood creatinine |
| HE_dbp | Final diastolic blood pressure (2nd and 3rd mean) |
| HE_dbp1 | Diastolic blood pressure (1st) |
| HE_dbp2 | Secondary diastolic blood pressure |
| HE_dbp3 | Diastolic blood pressure (3rd) |
| HE_fst | Fasting time |
| HE_glu | Glucose |
| HE_HB | Hemoglobin |
| HE_HBsAg | Hepatitis B surface antigen |
| HE_HCT | Hematocrit |
| HE_HDL_st2 | HDL-cholesterol |
| HE_ht | Height |
| HE_PLS | Pulse rate (15 sec) |
| HE_RBC | Red blood cells |
| HE_sbp | Final systolic blood pressure (2nd and 3rd mean) |
| HE_sbp1 | Systolic pressure (1st) |
| HE_sbp2 | Systolic pressure (2nd) |
| HE_sbp3 | Systolic pressure (3rd) |
| HE_TG | Triglyceride |
| HE_Uph | Urine pH |
| HE_Usg | Urine specific gravity |
| HE_WBC | White blood cells |
| HE_wc | Waist circumference |
| HE_wt | Weight |
| LQ_VAS | EuroQoL : VAS |
| LQ1_mn | Number of days of illness in the last month |
| LQ2_mn | Number of days of absence in the last month |
| N_B1 | Thiamin intake (mg) |
| N_B2 | Riboflavin intake (mg) |
| N_CA | Calcium intake (mg) |
| N_CAROT | Carotene intake (mg) |
| N_CHO | Carbohydrate intake (g) |
| N_EN | Energy intake (Kcal) |
| N_FAT | Fat intake (g) |
| N_FE | Iron intake (mg) |
| N_INTK | Dietary intake (g) |
| N_K | Potassium intake (mg) |
| N_NA | Sodium Intake (mg) |
| N_NIAC | Niacin intake (mg) |
| N_PHOS | Phosphorus intake (mg) |
| N_PROT | Protein intake (g) |
| N_RETIN | Retinol intake (μg) |
| N_VA | Vitamin A (retinol equivalent) intake (μgRE) |
| N_VITC | Vitamin C intake (mg) |
| N_WAT_C | Water intake (cup) |
| N_WATER | Water intake (g) |
| O_DMFTP | Caries experience permanent teeth number |
| tins | Type of health insurance |

Supplementary Table 3. Parameters of machine learning algorithms used to predict hazardous drinkers in K-NHANES datasets.

| **Classifier** | **Parameters** | | | |
| --- | --- | --- | --- | --- |
| **Support vector machine** | **Kernel function** | **Penalty** | **Loss function** |  |
|  | Linear | L2 | Square of hinge loss |  |
| **K-neighbors** | **Number of neighbors** | **Distance metric** | **Distance weight** | **Leaf_size** |
|  | 5 | minkowski | uniform | 30 |
|  | 5 | minkowski | distance | 30 |
|  | 10 | minkowski | uniform | 30 |
|  | 10 | minkowski | distance | 30 |
| **Random forest** | **Number of trees** | **Criterion** | **Min_samples_split**† | **Min_samples_leaf**‡ |
|  | 50 | gini | 2 | 1 |
|  | 50 | entropy | 2 | 1 |
|  | 100 | gini | 2 | 1 |
|  | 100 | entropy | 2 | 1 |
| **Logistic regression** | **Penalty** | **Solver** | **Max_iter**§ |  |
|  | L2 | lbfgs | 100 |  |
| †The minimun number of samples required to split an internal node ‡The minimum number of samples required to be at a leaf node §Maximum number of iterations taken for the solvers to converge. | | | | |

Supplementary Table 4. Comparison analysis using One-way ANOVA and Post-hoc analysis (Deep learning and conventional machine learning algorithms).

| **One-way ANOVA** | | | | | |
| --- | --- | --- | --- | --- | --- |
|  | Sum of squares | Degree of freedom | Mean square | F value | p-value |
| Between groups | 0.111 | 4 | 0.028 | 1606.006 | <0.001 |
| Within groups | 0.001 | 45 | <0.001 |  |  |
| Total | 0.112 | 49 |  |  |  |
| **Post-hoc analysis (Bonferroni)** | | | | | |
| Variable 1 | Variable 2 | Mean difference | Standard deviation | p-value | 95% CI |
| Deep learning | Logistic regression | 0.011 | 0.002 | <0.001 | 0.006-0.017 |
|  | Random forest | 0.060 | 0.002 | <0.001 | 0.054-0.065 |
|  | Linear SVM | 0.020 | 0.002 | <0.001 | 0.015-0.026 |
|  | K-nearest neighbors | 0.130 | 0.002 | <0.001 | 0.124-0.135 |

Supplementary Table 5. Comparison of performance metrics of classification algorithms.

|  | AUC | Accuracy | Precision | True positive rate | False positive rate | F1-score |
| --- | --- | --- | --- | --- | --- | --- |
| Deep learning | 0.870 | 0.822 | 0.756 | 0.624 | 0.090 | 0.684 |
| Logistic regression | 0.858 | 0.808 | 0.746 | 0.570 | 0.086 | 0.646 |
| Linear SVM | 0.849 | 0.791 | 0.649 | 0.705 | 0.170 | 0.676 |
| Random forest classifier | 0.810 | 0.765 | 0.668 | 0.475 | 0.105 | 0.555 |
| K-nearest neighbors | 0.740 | 0.732 | 0.589 | 0.432 | 0.134 | 0.498 |
